# Supplementary material for: Distinct patterns of brain activity mediate perceptual and motor and autonomic responses to noxious stimuli
Source: Nat Commun. 2018 Oct 26;9:4487. doi: 10.1038/s41467-018-06875-x (PMC6203833; doi:10.1038/s41467-018-06875-x)
Supplement: Supplementary file 1 — Supplementary Information [file 41467_2018_6875_MOESM1_ESM.pdf]

Supplementary Material for

Distinct patterns of brain activity mediate perceptual and motor and autonomic responses to noxious stimuli

Tiemann et al.

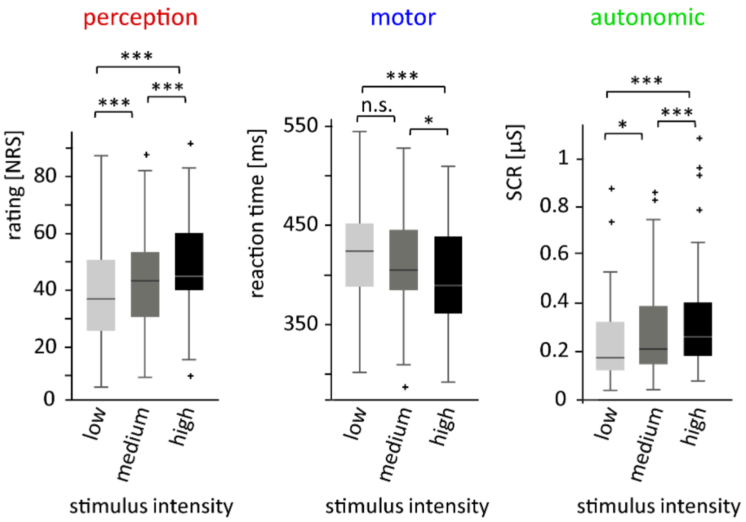

Supplementary Figure 1. Perceptual, motor and autonomic responses to noxious stimuli. Box plots of pain ratings (0-100, NRS), reaction times (ms) and skin conductance responses (µS) to noxious stimuli of low, medium and high intensity in the *combined* condition. The band inside the box indicates the median, and the bottom and top edges of the box indicate the 25th and 75th percentiles, respectively. The whiskers extend to the most extreme data points not considered outliers, and the outliers are plotted individually using the '+' symbol. ***p* < 0.01, ****p* < 0.001. n.s., not significant. NRS, numerical rating scale; SCR, skin conductance response. One-way repeated-measures analyses of variance (ANOVAs) showed that noxious stimulus intensity significantly influenced pain ratings, reaction times and SCRs (perception: $F_{(2,74)} = 58.02$, $p < 0.001$; motor: $F_{(2,74)} = 11.74$, $p < 0.001$; autonomic: $F_{(1, 52)} = 17.84$, $p < 0.001$). Pain ratings (low: 39 ± 19, medium: 45 ± 18, high: 49 ± 18; mean ± SD) and SCRs (low: 0.24 ± 0.19 µS, medium: 0.29 ± .22 µS, high: 0.35 ± 0.25 µS) increased with increasing stimulus intensity whereas reaction times decreased (low: 418 ± 58 ms, medium: 409 ± 56 ms, high: 396 ± 53 ms). Posthoc pairwise comparisons confirmed that all outcome measures differed significantly between stimulus intensities (paired sample *t*-tests, all $p < 0.02$) with the exception of a non-significant difference between the reaction times at low and medium stimulus intensities ($t_{(37)} = -1.92$, $p = 0.19$). Pearson correlation coefficients averaged across individuals were r = -0.24 for pain ratings and reaction times, r = -0.04 for reaction times and SCR, and r = 0.27 for pain ratings and SCR, corresponding to small effect sizes. The mean coefficient of determination (r^2) averaged across individuals was 9% ± 10 of shared variance between pain ratings and reaction times, 4% ± 4 between reaction times and SCRs, and 13% ± 11 between pain ratings and SCRs.

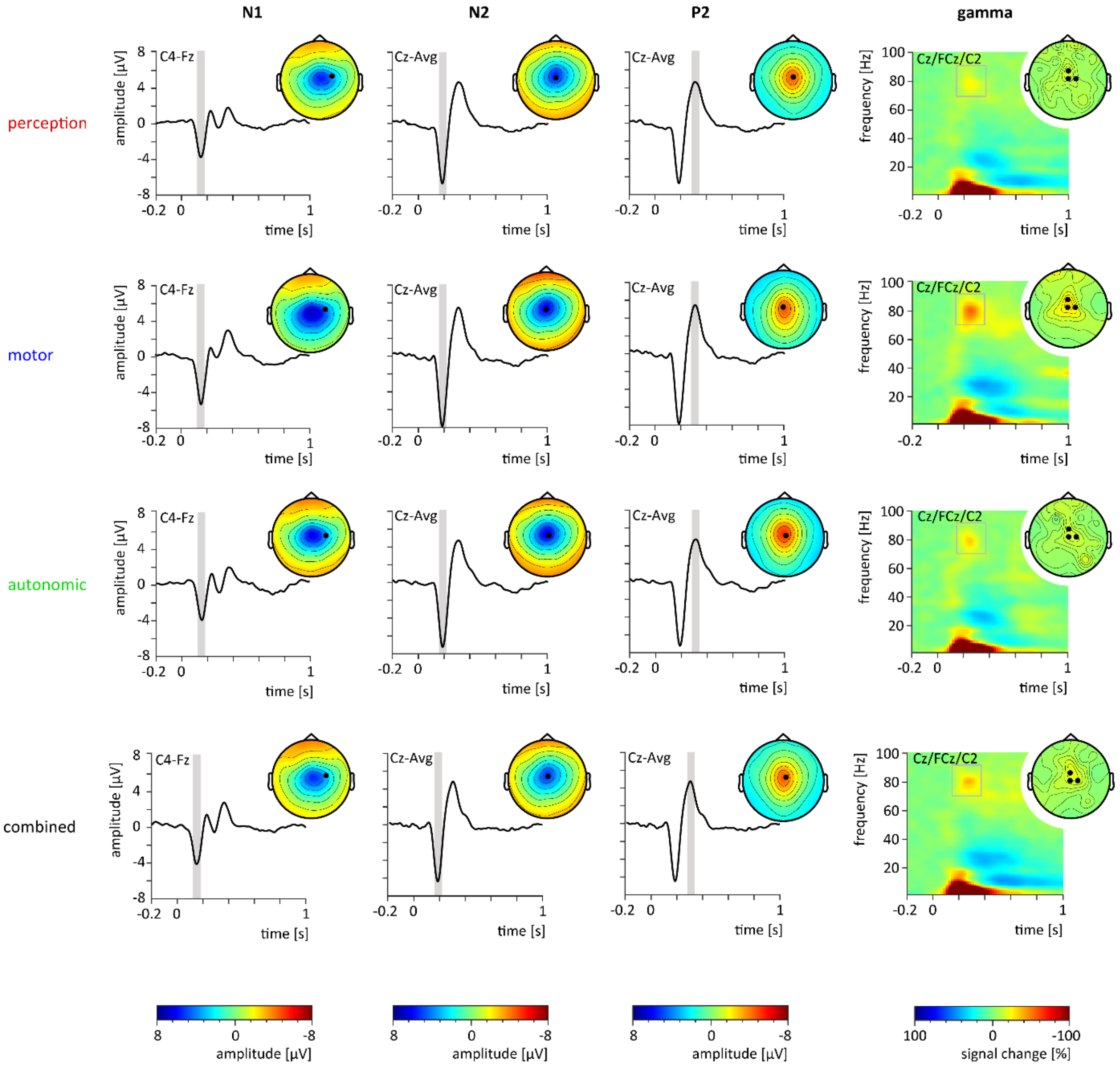

Supplementary Figure 2. Brain responses to noxious stimuli in the *perception*, *motor* and *autonomic* conditions. Mean time courses and time-frequency representation (TFR, right panel) of brain responses averaged participants for each condition. Marked time periods and time-frequency windows indicate periods/windows chosen to quantify N1, N2, P2 and gamma responses. Topographies depict the scalp distribution of neural activity in these periods/windows, electrodes used for the quantification of the different responses are marked. For visualization only, the TFR is displayed as %-signal change relative to a prestimulus baseline (-1000 to 0 ms).

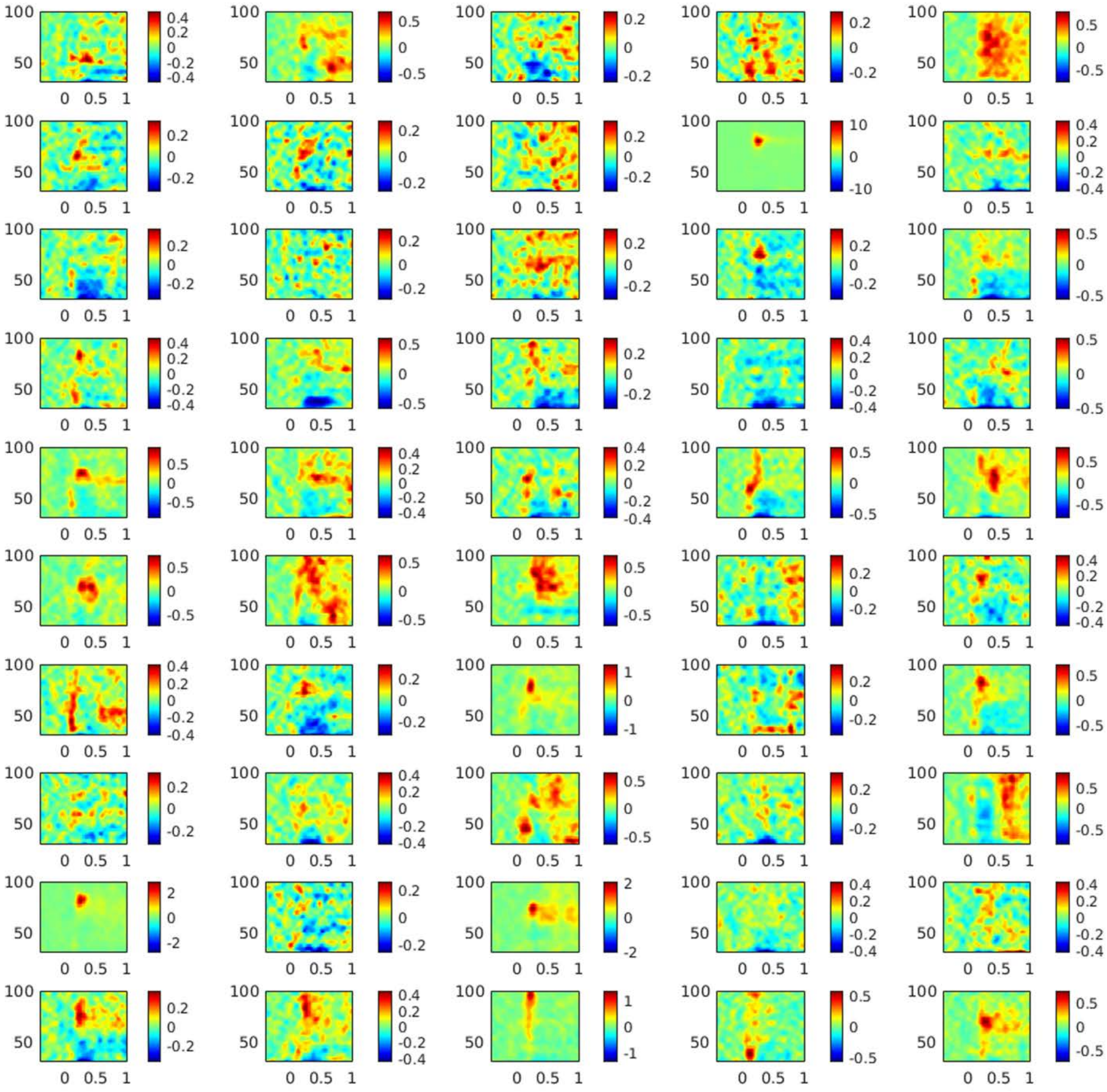

Supplementary Figure 3. Individual (n = 50) time-frequency representations (TFRs) calculated on the concatenated data from all four conditions (*perception*, *motor*, *autonomic*, *combined*), displayed for the time period from -400 - 1000 ms and the frequency range from 30 - 100 Hz. Power values are scaled to the individual maximum.

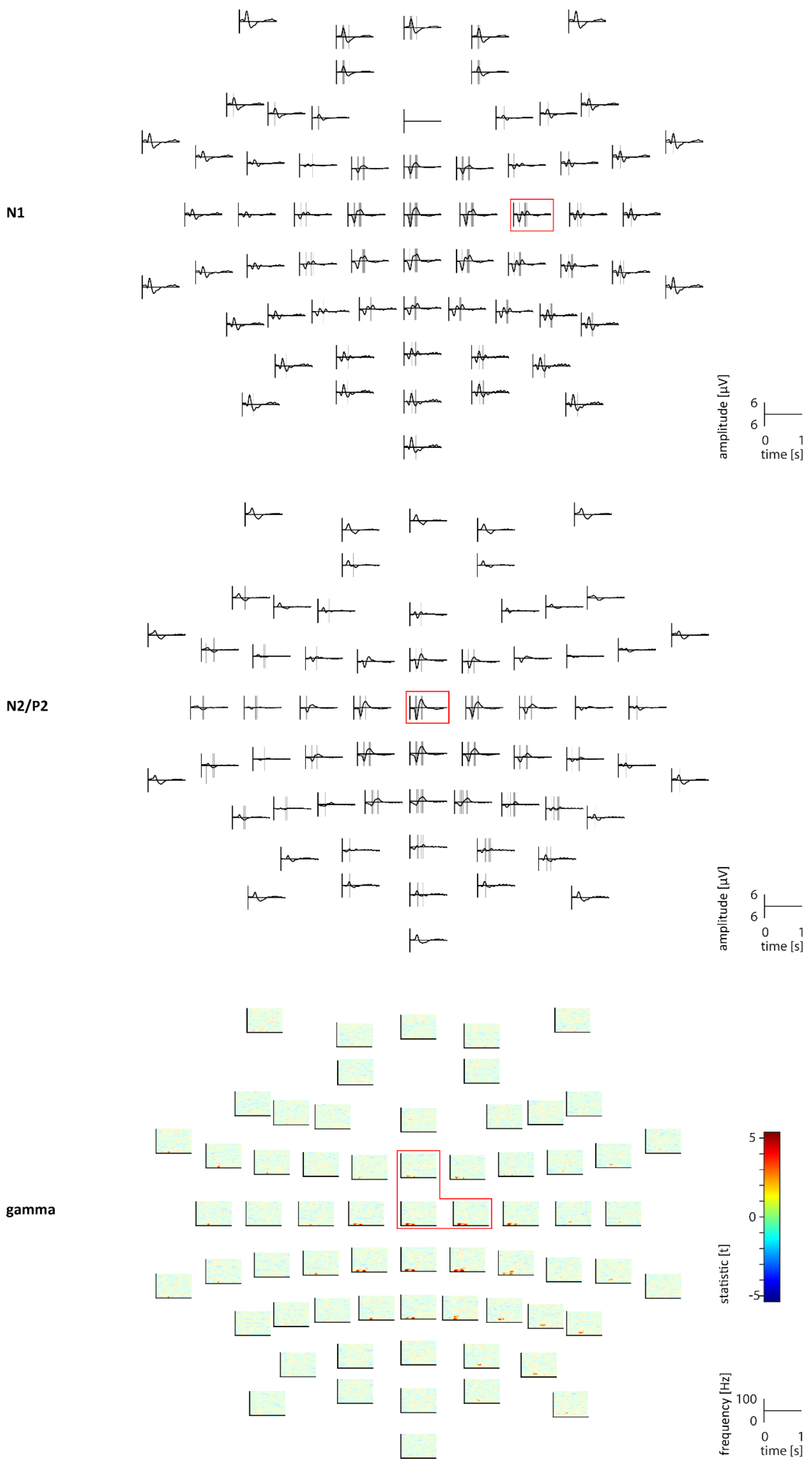
N1
amplitude [µV]
6
6
0
1
time [s]
N2/P2
amplitude [µV]
6
6
0
1
time [s]
gamma
statistic [t]
5
0
-5
frequency [Hz]
100
0
0
1
time [s]

Supplementary Figure 4. Grand-average evoked potential time courses and time-frequency representations (TFRs) of brain responses at all electrodes in the *perception* condition. Red boxes mark the electrodes which were used for analysis of N1, N2, P2 waves and gamma oscillations. Clusters of electrode-time(-frequency) points showing significant mediation effects in the relationship between stimulus intensity and pain perception based on cluster-based permutation statistics are marked in grey (upper two panels) or opaque color (lower panel).

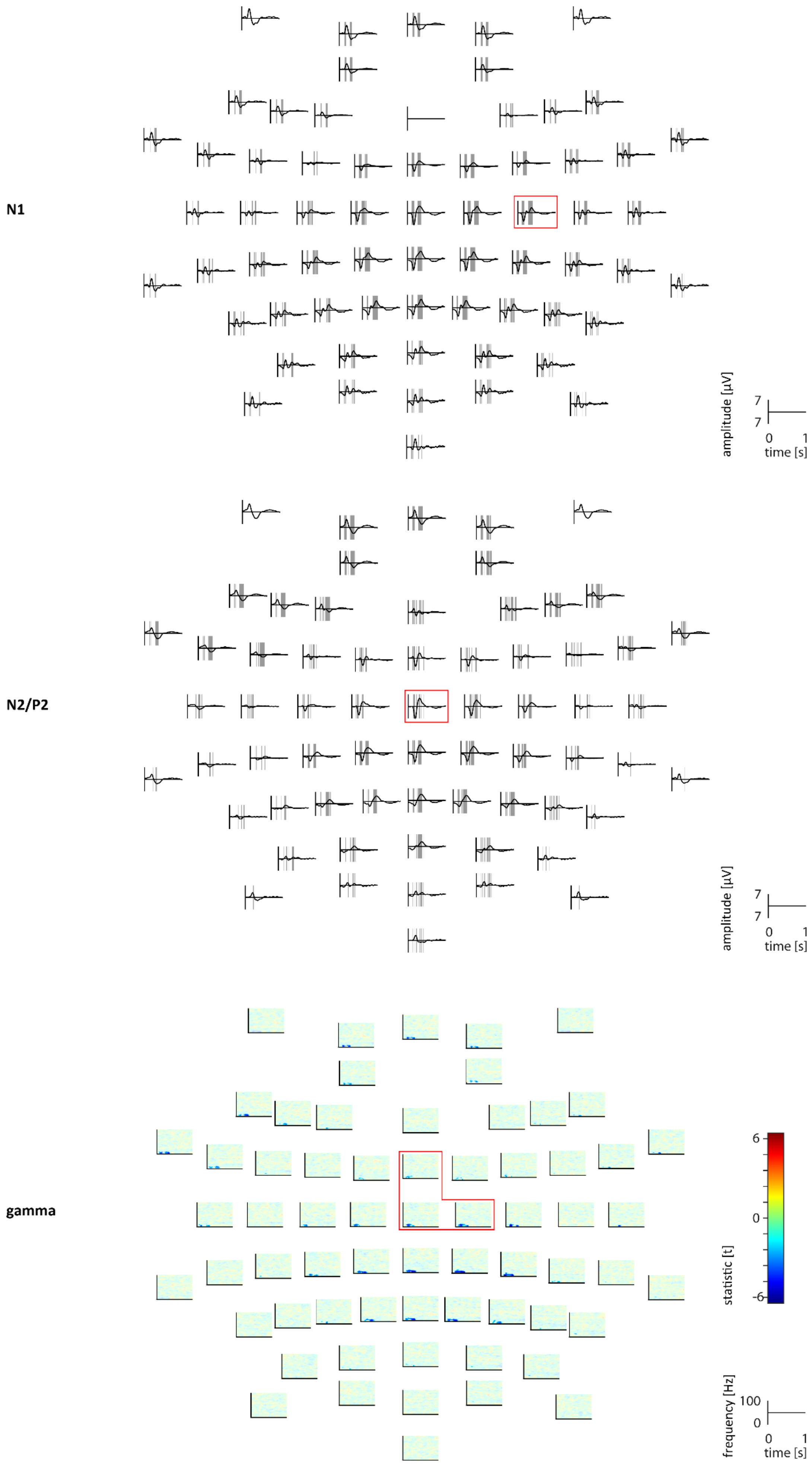
N1
amplitude [µV]
7
7
0 1
time [s]
N2/P2
amplitude [µV]
7
7
0 1
time [s]
gamma
statistic [t]
6
0
-6
frequency [Hz]
100
0
0 1
time [s]

Supplementary Figure 5. Grand-average evoked potential time courses and time-frequency representations (TFRs) of brain responses at all electrodes in the *motor* condition. Details are similar to Suppl. Fig. 4.

N1

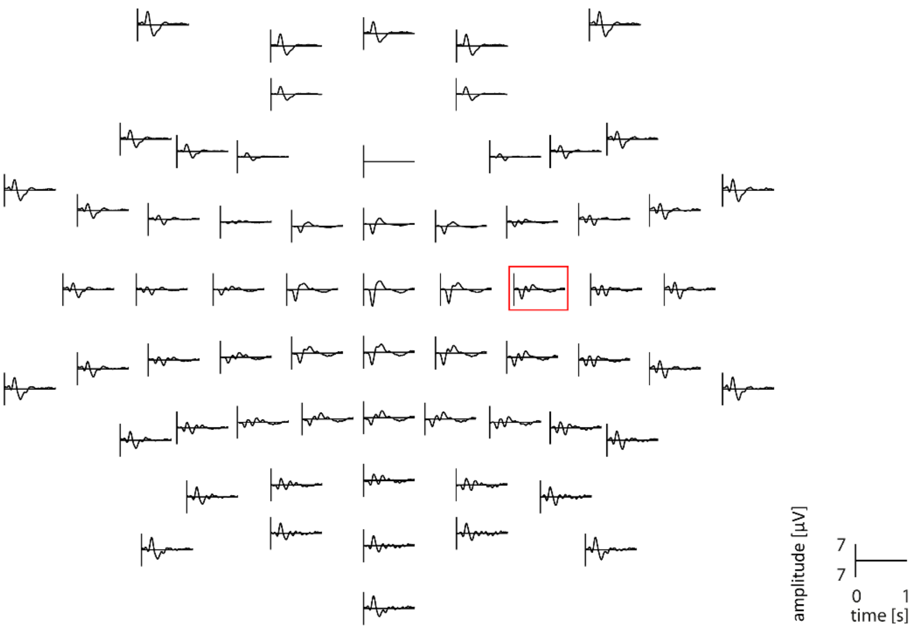

N2/P2

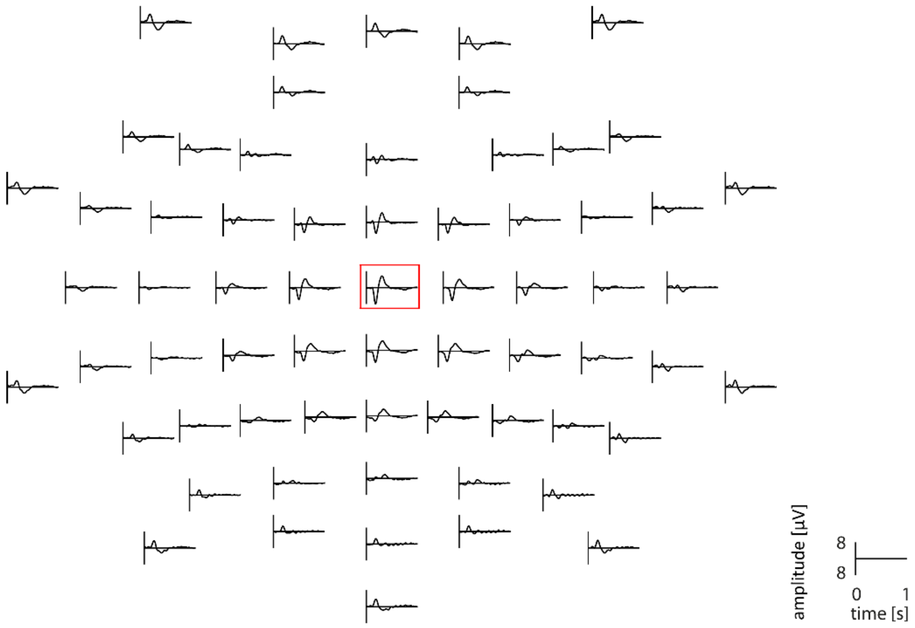

gamma

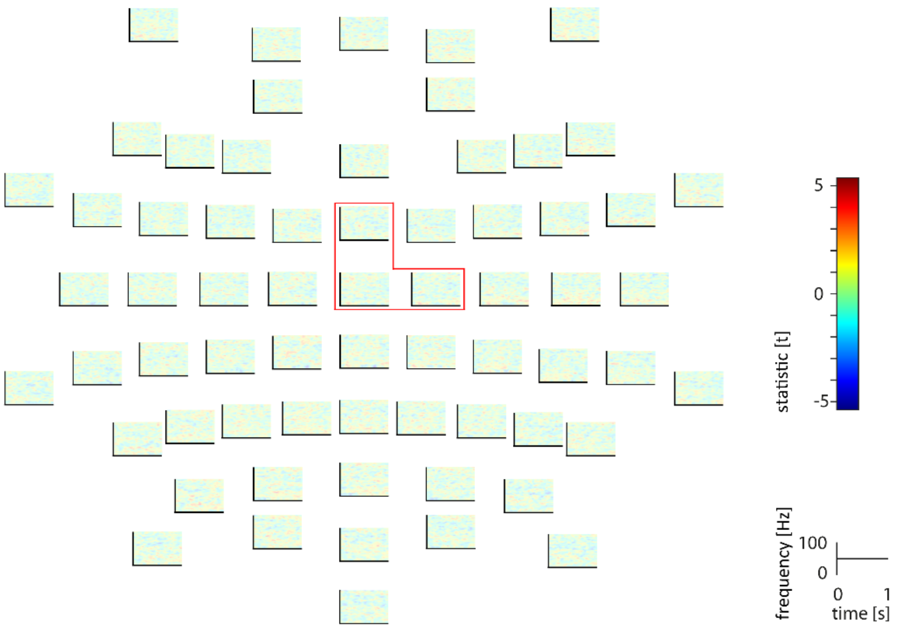

Supplementary Figure 6. Grand-average evoked potential time courses and time-frequency representations (TFRs) of brain responses at all electrodes in the *autonomic* condition. Details are similar to Suppl. Fig. 4.

		Perception				Motor				Autonomic			
		β	*SE*	*Z*	*p*	*β*	*SE*	*Z*	*p*	*β*	*SE*	*Z*	*p*
N1	a	.0068	.0178	.3541	.7233	.0068	.0176	.3939	.6936	.0068	.0177	.4017	.6879
	b	-.0920	.0188	-3.295	**.0013**	.1569	.0186	3.921	**.0004**	-.0421	.0111	-3.459	**.0005**
	c'	.1647	.0159	3.907	**.0001**	-.0833	.0196	-3.538	**.0005**	.0957	.0103	3.723	**.0002**
	c	.1636	.0162	3.982	**.0001**	-.0843	.0190	-3.580	**.0005**	.0977	.0103	3.728	**.0002**
	a*b	-.0003	.0012	-.3430	.9755	-.0011	.0021	-.5200	.6031	.0007	.0007	1.144	.2528
N2	a	.0108	.0218	.5069	.7233	.0108	.0216	.4784	.6936	.0108	.0218	.4720	.6879
	b	-.1101	.0226	-3.485	**.0010**	.0929	.0297	2.677	**.0149**	-.0943	.0180	-3.467	**.0005**
	c'	.1620	.0155	4.098	**.0001**	-.0784	.0183	-3.668	**.0005**	.1007	.0103	3.672	**.0002**
	c	.1636	.0164	4.066	**.0001**	-.0843	.0189	-3.539	**.0005**	.0977	.0103	3.694	**.0002**
	a*b	.0000	.0017	-.0177	.9859	-.0018	.0023	-.8070	.5596	-.0023	.0014	-1.739	.1640
P2	a	.0586	.0199	3.105	**.0038**	.0586	.0201	3.157	**.0032**	.0586	.0199	3.162	**.0031**
	b	.0664	.0231	2.954	**.0031**	-.0811	.0342	-2.444	**.0194**	.0628	.0186	3.701	**.0004**
	c'	.1531	.0150	3.846	**.0001**	-.0747	.0184	-3.502	**.0005**	.0913	.0104	3.750	**.0002**
	c	.1636	.0164	4.089	**.0001**	-.0843	.0190	-3.518	**.0005**	.0977	.0104	3.732	**.0002**
	a*b	.0027	.0016	1.717	.1719	-.0035	.0026	-1.335	.5008	.0020	.0014	1.449	.1966
gamma	a	.0268	.0081	3.717	**.0008**	.0268	.0081	3.725	**.0008**	.0268	.0081	3.685	**.0009**
	b	.1179	.0321	3.513	**.0010**	-.0631	.0514	-1.203	.2289	.1580	.0329	4.016	**.0002**
	c'	.1569	.0157	3.839	**.0001**	-.0788	.0177	-3.648	**.0005**	.0906	.0103	3.775	**.0002**
	c	.1636	.0165	4.010	**.0001**	-.0843	.0189	-3.589	**.0005**	.0977	.0103	3.722	**.0002**
	a*b	.0020	.0011	1.891	.1719	-.0016	.0015	-1.149	.5008	.0022	.0011	2.188	.1146

Supplementary Table 1. Results of the two-path mediation analysis in the *combined* condition. The table shows the second-level statistics for the mediation analyses of N1, N2, P2 and gamma responses for the perceptual, motor and autonomic outcome measure in the *combined* condition. All *p*-values are FDR-corrected. Significant effects of paths a, b, c and c' are marked in bold. ß, regression coefficient; SE, standard error. Standard errors for path a*b coefficients were significantly higher in the *combined* compared to the *perception*, *motor* and *autonomic* conditions (two-tailed paired *t*-test, $t_{(11)} = -5.4$, $p < 0.001$). Moreover, the overall pattern of path a*b coefficients was not significantly different between *combined* and *perception*, *motor* and *autonomic* conditions (two-tailed paired *t*-test, $t_{(11)} = 0.07$, $p = 0.95$) but instead significantly correlated (Pearson correlation, r = 0.66, $p = 0.02$).

	Perception				Motor											
	N2-P2				N1-P2				N1 - gamma				gamma - P2			
	β	*SE*	*Z*	*p*	*β*	*SE*	*Z*	*p*	*β*	*SE*	*Z*	*p*	*β*	*SE*	*Z*	*p*
a1	-.048	.014	-3.18	**.002**	-.024	.018	-1.51	.132	-.024	.0172	-1.58	.132	.034	.007	3.05	**.007**
b2	-.069	.046	-1.60	.109	-.075	.026	-3.07	**.003**	-.045	.0137	-2.74	**.006**	.131	.030	3.08	**.003**
d	.104	.017	2.97	**.003**	-.078	.025	-3.14	**.003**	-.065	.0327	-1.77	.076	-.086	.027	-3.17	**.003**
c'	.162	.019	3.49	**.001**	-.124	.019	-2.87	**.006**	-.127	.0179	-2.94	**.006**	-.128	.020	-2.69	**.007**
c	.180	.017	3.31	**.001**	-.144	.018	-2.90	**.004**	-.144	.0169	-2.97	**.004**	-.144	.017	-2.85	**.004**
a1*d*b2	.000	.000	1.44	.151	-.001	.000	-0.91	.538	.000	.0000	.158	.874	-.000	.000	-1.37	.525

Supplementary Table 2. Results of the three-path mediation analysis. Given are the second-level statistics for the three-path mediation analyses for the N2-P2 mediator sequence in the *perception*, and the N1-P2, N1-gamma, and gamma-P2 mediator sequence in the *motor* condition. All *p*-values are FDR-corrected. Significant coefficients of paths a, b, c and c' are marked in bold. ß, regression coefficient; SE, standard error.
